# Supplementary material for: A blood- and brain-based EWAS of smoking
Source: Nat Commun. 2025 Apr 4;16:3210. doi: 10.1038/s41467-025-58357-6 (PMC11968855; doi:10.1038/s41467-025-58357-6)
Supplement: Supplementary file 2 — Description of Additional Supplementary Files [file 41467_2025_58357_MOESM2_ESM.pdf]

Description of Additional Supplementary Files

Supplementary Data 1. Cohort description.

Supplementary Data 2. Sites associated with smoking (pack years) at posterior inclusion probability (PIP)>80% in blood-based EWAS (n=17,865). The sample size, PMID and P value are reported for CpG sites present in the EWAS catalog. The columns "Found in previous Illumina 450k EWAS" and "Found in previous Illumina EPIC EWAS" indicate CpG sites identified in prior studies that used the Illumina 450k BeadChip array (N=15,907, PMID: 27651444) and the Illumina EPIC 850k array (N=15,014, PMID: 38199042), respectively.

Supplementary Data 3. Smoking EWAS results across DNA methylation quantification technologies. Tables A, B, and C display the overlap between CpG sites identified by each technology. For each set of significant sites, the tables show how many were also measured by the other technologies, how many passed quality control (QC), and how many remained significant (P<1x10-5).

Supplementary Data 4. Sites associated with smoking status at P<1x10-5 in blood-based EWAS. DNA methylation was profiled using TWIST human methylome panel (n=46).

Supplementary Data 5. Sites associated with smoking status at P<1x10-5 in blood-based EWAS. DNA methylation was profiled using Oxford Nanopore Sequencing (n=46).

Supplementary Data 6. Gene set enrichment analysis results for TWIST hits at P<1x10-5 in blood-based EWAS, as returned by FUMA.

Supplementary Data 7. Elastic-net derived coefficients for mCigarette – a DNAm biomarker of cigarette consumption

Supplementary Data 8. Replication of mCigarette in ALSPAC. Time codes correspond to the following subsets of ALSPAC volunteers: antenatal - mothers, FOM_FOF - Focus on Mothers

and Focus on Fathers at midlife, F17 and F24 - offspring aged approximately 17 and 24 years old, respectively.

Supplementary Data 9. CpG sites associated with smoking status in 5 regions of brain tissue (n=14).

Supplementary Data 10. Sites associated with self-reported (pack years) and epigenetic (Grimage pack years) smoking above suggestive threshold of P<10-5 in blood-based GWAS (n=17,105). The results of analyses conducted in GS were compared to the largest GWAS of smoking pack years conducted to date (Erzurumluoglu et al., 2020). Genome Reference Consortium Human Build version (all experiments): GRCh37.

Supplementary Data 11. Lead loci associated with epigenetic (Grimage pack years) smoking above P<5x10-8 in blood-based GWAS (n=17,105). Genome Reference Consortium Human Build version: GRC37.

Supplementary Data 12. GWAS catalogue lookup results for the lead loci associated with Grimage pack years in blood-based GWAS (n=17,105). Genome Reference Consortium Human Build version: GRC37.

Supplementary Data 13. GoDMC lookup results for the lead loci associated with Grimage pack years in blood-based GWAS (n=17,105). Genome Reference Consortium Human Build version: GRC37.

Supplementary Data 14. The comparison of GrimAge DNAm pack years GWAS results to previously published GWAS studies of tobacco use (Saunders et al., 2022). SNPs were filtered to loci significant at P<5x10-8. Grimage DNAm pack years GWAS used Genome Reference Consortium Human (GRCh) Build version 37. GWASs from Saunders et al. were converted from GRCh38 to CRCh37 for compability.

Supplementary Data 15. DNA methylation processing and quality control information for Generation Scotland and the Lothian Birth Cohort 1936.

Supplementary Data 16. DNA processing and quality control information for Generation Scotland.

Supplementary Data 17. Sites associated with smoking (pack years) at posterior inclusion probability (PIP)>80% in blood-based EWAS (n=17,865) corrected for white blood cell proportions.
